# Characterization of Chickpea Seed Oil and Its Structuring Into Oleogels Using Rice Bran Wax: A Study on the Physicochemical, Thermal, Textural, and Antioxidant Properties for Potential Use in Health‐Conscious and Sustainable Food Products

**DOI:** 10.1002/fsn3.71511

**Published:** 2026-02-08

**Authors:** Farhang Hameed Awlqadr, Othman Abdulrahman Mohammed, Syamand Ahmed Qadir, Ako Mahmood Qadir, Aryan Mahmood Faraj, Khaled Arab

**Affiliations:** ^1^ Food Science and Quality Control, Halabja Technical College Sulaimani Polytechnic University Sulaymaniyah Iraq; ^2^ Medical Laboratory Science Department, Halabja Technical College Sulaimani Polytechnic University Sulaymaniyah Iraq; ^3^ Medical Laboratory Techniques Department, Halabja Technical Institute, Research Center Sulaimani Polytechnic University Sulaymaniyah Iraq; ^4^ Department of Food Science and Technology, Faculty of Agriculture University of Tabriz Tabriz Iran

**Keywords:** chickpea seed oil, functional properties, lipid structuring, oleogel, rice bran wax, sustainable fat replacers

## Abstract

The growing health concerns associated with saturated and trans fats have increased the demand for natural, plant‐based fat alternatives with functional and nutritional benefits. Oleogels, formed by structuring liquid oils with gelators, offer a promising strategy to mimic the physical properties of solid fats while preserving the healthful qualities of unsaturated oils. In this study, chickpea ( *Cicer arietinum* L.) seed oil—rich in polyunsaturated fatty acids such as linoleic and oleic acids—was explored as a base oil for oleogelation using rice bran wax (RBW), a natural, sustainable structuring agent. The oil was extracted using Soxhlet extraction and analyzed for its fatty acid composition and key physicochemical properties. Oleogels were developed using three RBW‐to‐oil ratios (3:7, 2:8, and 1:9) and assessed for oil binding capacity, antioxidant activity, thermal behavior, texture, structural integrity, and antimicrobial potential. Among the tested formulations, the 3:7 RBW‐to‐oil ratio demonstrated the most favorable performance in terms of oil retention, oxidative stability, and mechanical strength. Structural characterization via FTIR and XRD confirmed the formation of a physically structured gel network, while DSC analysis revealed stable thermal behavior. Antioxidant and antimicrobial assays indicated that all oleogels retained bioactivity, with differences depending on the wax content. The study confirms that RBW is an effective gelator for chickpea oil, enhancing its functionality and shelf stability. These findings highlight the potential of chickpea oil‐based oleogels, particularly the 3:7 formulation, for use in health‐oriented food products such as spreads, bakery fats, and meat alternatives, offering a sustainable and nutritionally improved replacement for conventional solid fats.

## Graphical abstract

Higher rice bran wax ratios produced firmer, more stable oleogels. Lower wax concentrations resulted in weak structuring and phase separation. Oil‐binding capacity increased noticeably with RBW content. Thermal and textural properties improved in proportion to wax concentration. Overall, RBW–chickpea oil oleogels show strong potential as plant‐based fat replacers.

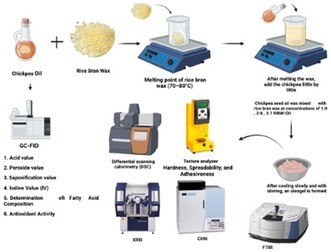

## 1 Introduction

The rising global awareness of diet‐related health issues has fueled the demand for alternatives to saturated and trans fats in food systems. Overconsumption of these fats is closely linked to cardiovascular diseases, obesity, and metabolic syndromes, prompting health authorities to recommend reducing their intake (Sacks et al. 2017). In response, researchers have increasingly explored plant‐based fat substitutes that can maintain desirable technological and sensory properties while improving nutritional profiles. Oleogels—semi‐solid systems formed by structuring liquid oils with low concentrations of gelators—have emerged as innovative fat replacers, mimicking the rheological, thermal, and textural properties of solid fats without compromising the health benefits of unsaturated oils (Wijarnprecha et al. 2018; Wang et al. 2023). Among underutilized plant oils, chickpea ( *Cicer arietinum* L.) seed oil has shown promise due to its high content of polyunsaturated fatty acids, particularly linoleic (~53%), oleic (~20%), and alpha‐linolenic acids (~12%) (Salaria et al. 2023). It also contains bioactive compounds such as tocopherols and phytosterols, which contribute to its antioxidant potential and health benefits (Jukanti et al. 2012). Despite this, chickpea oil is largely underutilized in functional food systems due to its high susceptibility to oxidation, which limits its shelf life and sensory acceptability. Stabilizing such oils requires an effective structuring agent capable of enhancing oxidative stability while preserving nutritional value. Rice bran wax (RBW), a by‐product of rice milling, is a sustainable, low‐cost, and natural gelator composed primarily of long‐chain saturated fatty alcohols and esters. RBW has demonstrated strong gelation ability even at low concentrations (2%–3%) and is known to produce thermoreversible, stable oleogels with excellent oil‐binding and structural properties (Wijarnprecha et al. 2018; Zhao et al. 2020). Several studies have shown the successful use of RBW in structuring oils such as soybean, sunflower, and corn oil, improving their oxidative stability, mechanical strength, and suitability for applications in baked goods, spreads, and meat substitutes (Jones et al. 2022; Hwang et al. 2022). However, there remains a critical research gap: the potential of chickpea seed oil to be structured into oleogels using RBW has not yet been adequately explored. Most existing studies have overlooked chickpea oil despite its nutritional benefits, and no systematic investigation has been conducted on how varying RBW‐to‐oil ratios influence the physicochemical, functional, and antimicrobial properties of such oleogels. Furthermore, while the oxidative stability and texture of RBW‐based oleogels have been studied in other oils, their performance with chickpea oil, particularly in terms of crystallinity, antioxidant capacity, and antimicrobial effectiveness, remains unknown.

The present study aims to fill this gap by developing chickpea seed oil‐based oleogels structured with RBW and evaluating their physicochemical characteristics, antioxidant and antimicrobial activity, thermal and structural properties, and oil‐binding capacity across three different wax‐to‐oil ratios (3:7, 2:8, and 1:9). The outcomes of this research will provide novel insights into the functional potential of chickpea oil oleogels, contributing to the development of sustainable, health‐promoting fat alternatives in food systems.

## 2 Material and Methods

### 2.1 Materials

Seeds of chickpea (*Cicer arietinum L*.) were obtained from the local market, and seed samples were thoroughly cleaned for dust and impurities. Products: Rice bran wax (RBW) was kindly provided by a certified food‐grade manufacturer. Chemicals and reagents: were analytical grade solvents such as hexane and ethanol and reagents for fatty acid analysis (potassium hydroxide, methanol) (Sigma‐Aldrich, or equivalent).

### 2.2 Extraction of Chickpea Seed Oil

**FIGURE 1:**
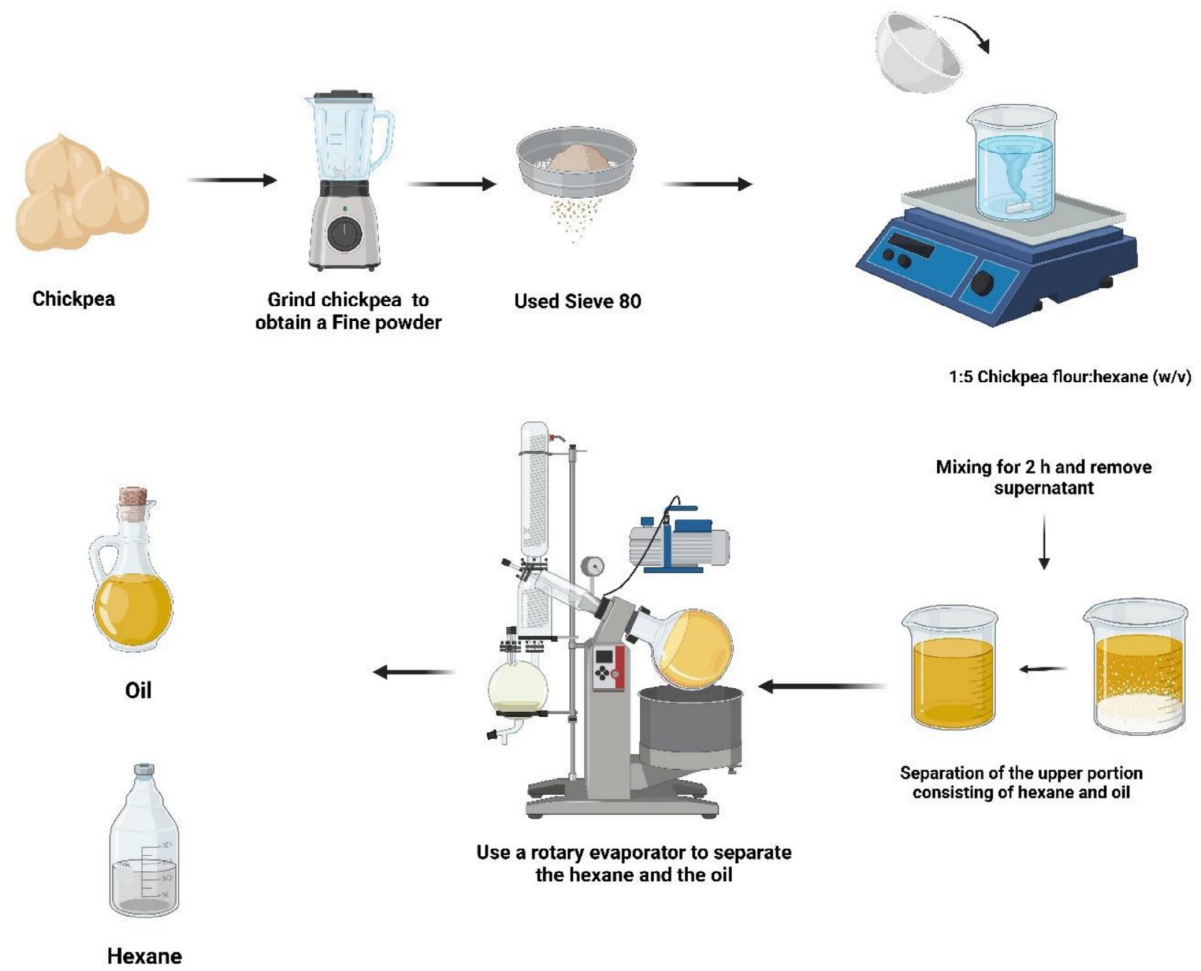
Method extraction oil in chickpea.

Chickpea ( *Cicer arietinum* L.) seeds were cleaned, dried, and ground to a fine powder before extraction. Oil was extracted using n‐hexane at a seed‐to‐solvent ratio of 1:5 (w/v). The mixture was thoroughly agitated and allowed to stand to ensure efficient penetration of the solvent into the seed matrix. The extract was then filtered, and the solvent was removed using a rotary evaporator under reduced pressure at low temperature to prevent thermal degradation. This process ensured complete elimination of residual hexane, yielding a clean, solvent‐free oil suitable for further physicochemical and functional analyses. This extraction approach is consistent with previously reported methods for seed oils (Ofori et al. 2023). As illustrated in Figure 1, the process flow of chickpea ( *Cicer arietinum* L.) seed oil extraction is presented.

### 2.3 Preparation of Rice Bran Wax Oleogels

**FIGURE 2:**
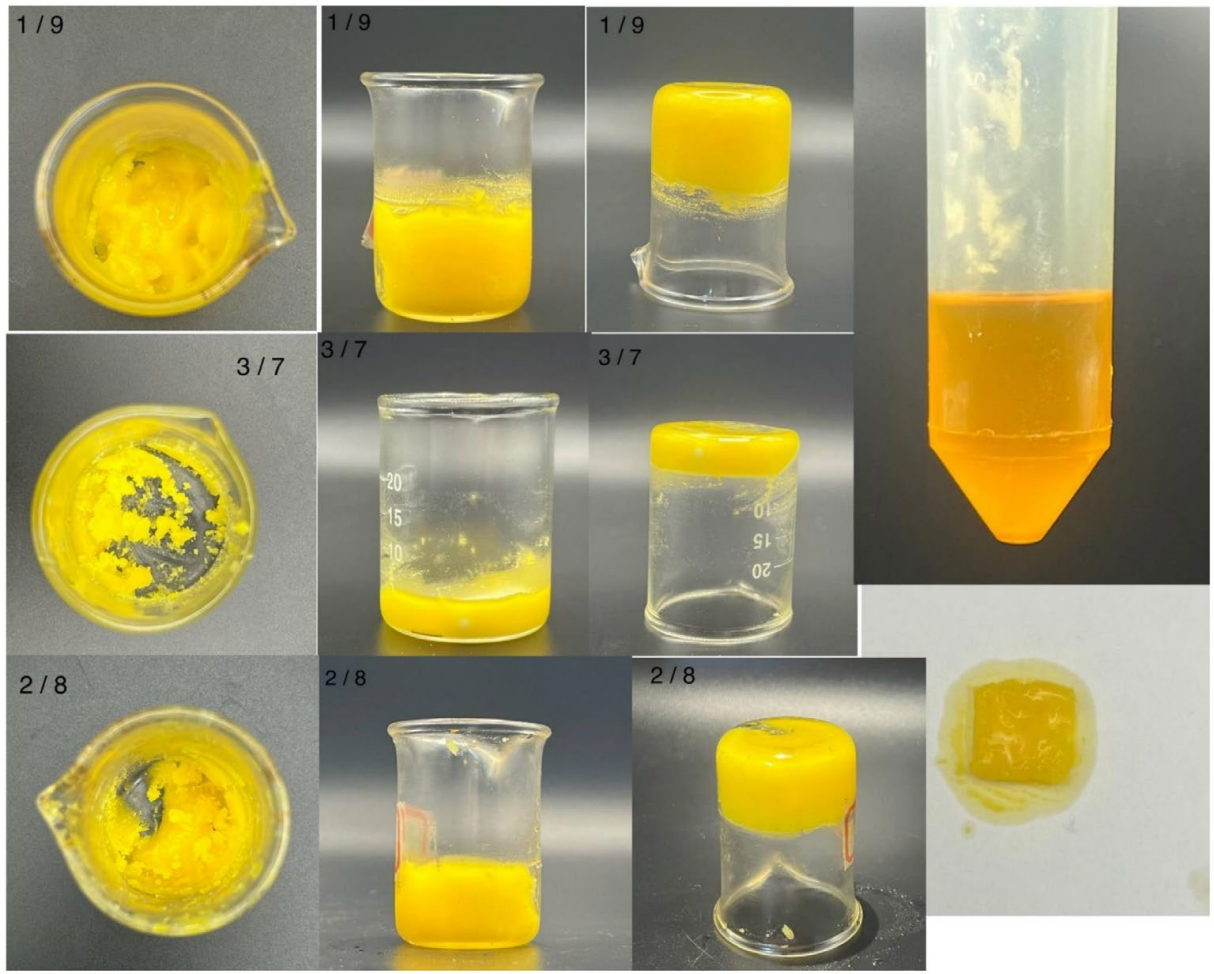
Visual characterization of oleogels prepared using chickpea oil and rice bran wax (RBW) at varying ratios: 1:9, 2:8, and 3:7 (RBW:Oil).

Oleogels were prepared using chickpea seed oil structured with rice bran wax (RBW) at three concentrations: 1:9, 2:8, and 3:7 (wax: oil, w/w). The visual appearance and structural differences among the three oleogel formulations are shown in Figure 2. RBW was weighed and added to the oil, and the mixture was heated to 70°C with continuous stirring to ensure complete melting and homogenization. Once fully dissolved, the hot mixture was poured into molds and allowed to cool at room temperature for 30 min. After cooling, they were refrigerated for 24 h until gelation was achieved. Oleogels were stored in airtight light protected containers to avoid oxidation prior to analysis. For each formulation (3:7, 2:8, and 1:9 RBW:oil), three independent batches were prepared on separate days to ensure reproducibility. All analytical measurements were performed in triplicate for each batch, and results are reported as mean ± standard deviation.

### 2.4 Physicochemical Characterization of Chickpea Oil

Complete physical and chemical characterization of chickpea oil is necessary to understand its quality, stability, and the possibility of its application in food systems such as oleogels. Acid value, peroxide value, saponification value, iodine value, and fatty acid composition were the main parameters analyzed in this study. These indices indicate the oxidative stability, molecular structure, and nutritional quality of oil.

#### 2.4.1 Acid Value (AV) and Free Fatty Acids (FFA)

The acid value was determined according to AOAC Official Method 940.28. A weighed chickpea oil sample (approximately 5 g) was dissolved in a neutralized ethanol‐diethyl ether solution (1:1, v/v) that was slightly warmed to assist in solubilizing the oil. The solid was titrated with 0.1 N potassium hydroxide (KOH) with phenolphthalein indicator until the sample remained pale pink according to this procedure (Gjoshevska et al. 2022). The acid value was determined using the following equation:

$$\mathrm{AV}=\frac{V\times N\times 56.1}{W}$$

where *V* = KOH use (mL), N = normality of KOH, *W* = weight of oil sample (g), 56.1 = M.wt KOH.

Free fatty acid (% oleic acid) was calculated as:

$$\mathrm{FFA}\;\left(\%\right)=\mathrm{AV}\times 0.503$$

#### 2.4.2 Peroxide Value (PV)

The peroxide value was determined using the iodometric titration method based on AOAC Official Method 965.33 (Zhang et al. 2021). Approximately 5 g of chickpea oil was dissolved in a mixture of glacial acetic acid and chloroform (3:2, v/v), followed by the addition of saturated potassium iodide (KI) solution 0.5 mL. The mixture was kept in the dark for 1 min, diluted with distilled water, and titrated with 0.01 N sodium thiosulfate solution using starch as an indicator. The PV was expressed in milliequivalents of active oxygen per kilogram of oil (meq O_2_/kg) and was calculated as

$$\mathrm{PV}\left(\frac{\mathrm{meq}O_{2}}{\mathrm{kg}}\right)=\frac{S\times N}{W}\times 1000$$

where *S* = Na_2_S_2_O_3_ used (mL), N = normality of Na_2_S_2_O_3_, *W* = weight of sample (g).

#### 2.4.3 Determination of Iodine Value (IV)

The iodine value was measured using the Wijs method (AOAC Official Method 993.20) (Samanta et al. 2023). Approximately 0.5 g of oil was dissolved in 20 mL of chloroform, followed by the addition of 25 mL of Wijs reagent (iodine monochloride solution). The mixture was kept in the dark for 30 min, after which 20 mL of a 10% potassium iodide solution and 100 mL of distilled water were added. The liberated iodine was titrated with 0.1 N sodium thiosulfate using starch as an indicator. A blank titration was performed. The iodine content was calculated as follows:

$$\mathrm{IV}=\frac{\left(B-S\right)\times N\times 12.69}{W}$$

where *B* = volume of Na_2_S_2_O_3_ for blank (mL), *S* = volume for sample (mL), N = normality of Na_2_S_2_O_3_, *W* = weight of oil sample (g), 12.69 = milliequivalent of iodine (g) per 100 g of oil.

#### 2.4.4 Determination of Saponification Value (SV)

Saponification value was determined according to AOAC Official Method 920.160 (Dalla Nora et al. 2018). A known weight of oil (approximately 2 g) was refluxed with 25 mL of 0.5 N alcoholic potassium hydroxide solution for 60 min. After cooling, the excess KOH was titrated with 0.5 N hydrochloric acid using phenolphthalein as an indicator. Blank titration was performed under identical conditions without oil. SV was calculated as follows:

$$\mathrm{SV}=\frac{\left(B-S\right)\times N\times 56.1}{W}$$

where *B* = V.HCl for blank (mL), *S* = V.HCl for sample (mL), N = normality of HCl, *W* = weight of sample (g).

#### 2.4.5 Determination of Fatty Acid Composition

Fatty acid methyl esters (FAMEs) were prepared by transesterification according to the AOAC Official Method 996.06 (Ekici et al. 2024). Approximately 100 mg of chickpea oil was reacted with 2 mL of 0.5 N methanolic KOH solution and heated at 60°C for 10 min. After cooling, 2 mL of hexane was added to extract the FAMEs. The hexane layer was separated and filtered for gas chromatographic analysis. Gas chromatography was performed using a GC‐FID (Gas Chromatograph with Flame Ionization Detector) equipped with a capillary column BPX70 or equivalent, 30 m × 0.25 mm, 0.25 μm film thickness. The injector and detector temperatures were set at 230°C, with an oven program starting at 180°C for 1 min and increasing to 230°C at a rate of 2°C/min. Fatty acids were identified by comparing retention times with those of standard FAMEs, and the results were expressed as the percentage of total fatty acids.

### 2.5 Functional Property Analysis of Oleogels

Functional property evaluation the performance and potential suitability of the RBW‐chickpea oil oleogels (for use in foods) were examined by a panel of functional properties tests. The analysis included studies on OBC, textural properties and thermal properties. These parameters play a vital role in the structural integrity, stability, and application of oleogels in food product formulations especially in the products that need fat‐like consistency and thermal resistance.

#### 2.5.1 Oil Binding Capacity (OBC)

Oil binding capacity (OBC) was measured to evaluate the ability of the oleogel network to retain oil under centrifugal force. Approximately 5 g of each oleogel sample was centrifuged at 9000 rpm for 30 min at 25°C. The released oil was decanted and weighed, and OBC was calculated as the percentage of oil retained within the gel matrix. Increased RBW content resulted in a more cohesive crystalline network and higher OBC.

The Oil Binding Capacity was calculated using the formula:

$$\mathrm{OBC}\left(\%\right)=\frac{W_{i}\times W_{O}}{W_{I}}$$

where $W_{i}$: initial sample weight, $W_{o}$: weight of separated oil.

Analyses were performed in triplicate and the means were calculated. This procedure is a modification of the methods published (Wijarnprecha et al. 2018), ensuring consistency with prior research.

#### 2.5.2 Texture Analysis

The texture profile attributes of the oleogels, firmness and spreadability, were analyzed by a Texture Analyzer. XTplus, Stable Micro Systems, UK. Such parameters are crucial to mimic the sensory and mechanical responses of the gels during their manipulation and food processing. For all tests, oleogel samples were prepared using cylindrical molds and equilibrated at room temperature (25°C) for a minimum of 1 h before testing (Perța‐Crișan et al. 2023). A cylindrical stainless steel (5 mm diameter) probe was inserted into the gel surface at 1 mm/s to a set depth of 10 mm. The penetrating force was reported as firmness (N), and the spreadability was estimated from the area under the curve or lower resistance during penetration. All results were obtained in triplicate, and values represent mean ± SD. This approach is a model of application in which oleogels are spread or cut and mimics normal texture profile analysis setup used in lipid structuring research.

#### 2.5.3 Differential Scanning Calorimetry (DSC)

The thermal properties of the oleogels were evaluated by Differential Scanning Calorimetry (DSC) to study the melting and crystallization behavior of the gellants, which has consequences on the applicability of the gelled product in storage and processing. Approximately 10 mg of each oleogel was placed in an aluminum DSC pan with an empty pan as the reference. DSC analysis was performed using calibrated apparatus, where the sample was heated from 0°C to 100°C at a rate of 5°C/min under nitrogen flow (to avoid oxidation) (Sena et al. 2022). Thermograms were shot and endothermic peaks, which pertain to melting transitions of wax‐crystal and lipid constituents, were found. Parameters including onset temperature, peak temperature, and enthalpy change (Δ*H*, J/g) were studied to establish the crystallinity and thermal stability of the gel.

#### 2.5.4 Oil Migration Ability

Oil migration was assessed using a filter‐paper ring test. A 0.5 g sample of oleogel was placed at the center of a Whatman No. 1 filter paper and allowed to stand at 25°C for 24 h. The oil halo radius was measured, and the migration area was calculated. Lower migration distances in high‐wax samples indicated stronger structural integrity and more effective oil entrapment (Han et al. 2022). The migration distance (*R*) was measured by using a digital caliper, and the migration area was calculated by the formula.

$$\text{Migration area}\;\left({\mathrm{cm}}^{2}\right)=\pi \;\left(R^{2}–r^{2}\right)$$

where *R* = radius of the outer oil ring, *r* = initial radius of the applied gel (0.5 cm).

#### 2.5.5 X‐Ray Diffraction (XRD) Analysis

The crystalline structure and molecular packing of the rice bran wax–chickpea oil oleogels, which influence their texture, thermal properties, and mechanical properties, were studied by means of X‐ray diffraction (XRD). All oleogel samples were formed and allowed to stabilize for 24 h at 4°C, and a thin uniform layer of each sample was used in transmission mode on a glass sample holder. X‐ray analyses (Bruker D8 advance) were performed at 40 kV and 30 mA via Cu‐Kα radiation (λ = 1.5406 Å). Scanning was performed over the 2θ range from 5° to 40° at a scanning speed of 2°/min and step of 0.02°. Short‐spacing reflections, which are indicative of molecular packing within the crystal lattice and characteristic of orthorhombic (*β*') or hexagonal (*α*) crystal structures (Pang et al. 2022). were identified based on the analysis of diffractograms. The crystallinity was also determined in the different samples and compared, and it was observed that sharper peaks and increased intensity peaks were correlated with more orderly and closer packed wax crystal networks, which would result in stronger and more stable oleogels. This strategy mimics those used in previous oleogel studies developed with wax, and reveals the structural aspects of how rice bran wax organizes within the chickpea oil network to form a structured‐gel.

#### 2.5.6 Elemental Analysis Using CHN Analyzer

The chickpea oil and its oleogel samples were analyzed for elemental analysis (C, H and N content) using CHN analyzer model Elementar Vario EL III. Approximately 2–5 mg of the dried sample was weighed into a tin capsule and combusted at 1000°C in an O_2_‐enriched atmosphere. The generated gases (CO_2_, H_2_O, N_2_) were determined with a thermal electrical conductivity detector (Proctor et al. 2022). Elemental percentages were derived from the combustion data and the instrument was calibrated with an internal standard (e.g., acetanilide). All measurements were made in triplicates, and the results were presented as the weight percent of C, H, and N content; the result testifies to the organic composition and purity of the samples.

#### 2.5.7 Fourier Transform Infrared Spectroscopy (FTIR)

FTIR analysis–functional characterization of compounds using fourier transform infrared spectroscopy was performed in chickpea oil and oleogel samples. A small quantity of each sample was transferred to an ATR (Attenuated Total Reflectance) accessory for the FTIR spectrometer PerkinElmer Spectrum Two. All spectra were recorded in the range of 4000–400 cm^−1^ with a resolution of 4 cm^−1^ and 32 scans per sample. Characteristic absorption bands were interpreted showing bondings of C–H, C=O, and O–H, representing lipid structures and the possible interactions between oil and wax. The result served as confirmation for the accomplished gelation and chemical monitoring of the oleogels (Sena et al. 2022).

### 2.6 Antioxidant Activity

#### 2.6.1 DPPH Radical Scavenging Assay

The DPPH radical scavenging activity was measured using the method described by Gulcin and Alwasel (2023). A 0.1 mM DPPH solution in methanol was prepared fresh. A 1 mL aliquot of extract was added to 3 mL of DPPH solution and incubated in the dark at room temperature for 30 min. The decrease in absorbance was measured at 517 nm. The scavenging activity was calculated as a percentage of inhibition compared to a blank (Gulcin and Alwasel 2023).

$$\text{Inhibition}\;\left(\%\right)=\left(\frac{A_{\text{control}}-A_{\text{Sample}}}{A_{\text{control}}}\right)\times 100$$

where control is the absorbance of DPPH without sample, and sample is the absorbance with the test sample. All tests were performed in triplicate.

#### 2.6.2 ABTS Radical Cation Decolorization Assay

Using the ABTS radical scavenging assay, chickpea oil and oleogel samples were assessed for antioxidant capacity. To create the ABTS radical cation (ABTS^+·^), 7 mM ABTS solution was mixed with 2.45 mM potassium persulfate and left at room temperature for 12–16 h in the dark. The solution was diluted with ethanol to achieve an absorbance of 0.70 ± 0.02 at 734 nm. After mixing 1 mL of sample extract with 1 mL of ABTS^+·^ solution, the absorbance was measured at 734 nm after 6 min using a UV–Vis spectrophotometer (Mingle and Newsome 2020). The percent inhibition was calculated using the same formula as in the DPPH assay. All measurements were carried out in triplicate, and results were expressed as % inhibition or compared against a standard antioxidant, Trolox.

### 2.7 Antimicrobial Activity

Chickpea seed oil and rice bran wax‐based oleogels were tested for antibacterial efficacy against *Bacillus subtilis* , *Staphylococcus aureus* , and *E. coli* using agar well diffusion. Approximately 1 × 10^8^ CFU/mL was standardized from fresh bacterial cultures cultured in nutrient broth at 37°C. Each bacterial strain was swabbed onto Mueller‐Hinton Agar (MHA) plates. Agar wells (6 mm diameter) were filled with 100 μL of sterilized sample (chickpea oil or oleogels). Incubated plates at 37°C for 24 h yielded millimeter‐scale inhibitory zones, comparing positive (ciprofloxacin) and negative (DMSO) controls. Antibacterial activity was measured by inhibition zone size, with larger zones showing stronger effects.

### 2.8 Statistical Analysis

All experiments were conducted in three independent batches, each analyzed in triplicate. Data were expressed as mean ± standard deviation (SD). Statistical differences were evaluated using one‐way ANOVA, followed by Tukey's HSD post hoc test, using: IBM SPSS Statistics, Version 26.0. Significance level was set at *p* < 0.05.

## 3 Results and Discussion

### 3.1 Physicochemical Characterization of Chickpea Oil

#### 3.1.1 Acid Value and Free Fatty Acids

The acid value (AV) shown in Table 1 is a crucial indicator of the free fatty acid (FFA) content in fats and oils, reflecting the degree of hydrolytic rancidity and lipid degradation. In this study, the AV of chickpea seed oil was found to be 2.7, while the oleogel samples exhibited significantly lower AVs, particularly 1.1 for the 3:7 formulation, 1.13 for 2:8, and 2.58 for 1:9. These findings definitely show that increased amounts of RBW in the oleogels were reflected in lower hydrolytic activity in the oil, thus enhancing the oxidative stability. The lower AV in RBW oleogels is consistent with the report of Sahu et al. (2020) that reported that rice bran wax aids in the stabilization of oil‐based systems by creating a crystalline matrix around the oil that shields it from hydrolytic degradation. The study indicated that the AV of oleogels prepared from RBW and rice bran oil fatty acid distillate when exposed to oxidation was narrower than that of control oils, thus confirming the oil protective effect of the wax (Sahu et al. 2020). In addition, the fact that the AV gradually went up as you decreased the wax content in your sample (from 3:7 to 1:9) shows that the structure of the oleogel is retiring (losing solidity) and there is more of its frame open to the oil that is available for moisture and hydrolytic enzymes. Dimakopoulou‐Papazoglou et al. (2023) the contribution of CBW to the oxidative and hydrolytic stability of the oleogels originates from the long‐chain saturated esters of CBW, restricting the FFA liberation and reducing the AV during storage. In fact, the oleogel with the greatest wax concentration (3:7) displayed the lowest acid value, which is consistent with the broader crystalline network examination having better protection from hydrolytic attack. On the other hand, the 1:9 sample, with the lowest concentration of wax, also presented the highest AV, which was closer to that of free chickpea oil, and hence with a less protective structure. The physicochemical and functional properties of the oleogels are presented in Table 2.

**TABLE 1:** Fatty acid composition of chickpea seed oil as determined by GC‐FID.

Peak ID	Fatty acid	Retention time (min)	Concentration (%)	Common name
1	C14:0	8.45	2.350	Myristic acid
2	C16:0	10.05	8.266	Palmitic acid
3	C18:1 (9)	14.05	19.917	Oleic acid (ω‐9)
4	C18:2 (9,12)	15.14	52.998	Linoleic acid (ω‐6)
5	C18:3 (9,12,15)	16.11	12.343	Alpha‐linolenic acid (ω‐3)
6	C20:0	17.35	4.274	Arachidic acid
7	C22:0	19.62	4.003	Behenic acid
8	C24:0	21.33	5.565	Lignoceric acid

**TABLE 2:** The key physicochemical and functional properties of the oleogels.

Sample	Acid value (mean ± SD)	FFA % (mean ± SD)	Peroxide value (PV) (mean ± SD)	Iodine value (IV) (mean ± SD)	Saponification value (SV) (mean ± SD)	DPPH (mean ± SD)	ABTS (mean ± SD)	Oil Binding capacity (mean ± SD)	Oil migration ability (mean ± SD)	*p* (ANOVA)	Post hoc test (Tukey's HSD)
Chickpea seed oil	2.7 ± 0.2^a^	1.36 ± 0.1^a^	2.1 ± 0.1^b^	114 ± 2^a^	190 ± 3^b^	43.79 ± 1.5^a^	35.53 ± 1.3^b^	—	—	—	—
Rice bran wax oleogel 3:7	1.1 ± 0.1^c^	0.55 ± 0.05^c^	1.8 ± 0.2^c^	110 ± 1^c^	188 ± 2^c^	39.82 ± 1.2^b^	15.75 ± 0.5^c^	96.0 ± 2.0^a^	16.4 ± 1.0^c^	< 0.05	3:7 < 1:9, 3:7 < 2:8
Rice bran wax oleogel 2:8	1.13 ± 0.1^c^	0.57 ± 0.06^c^	3.5 ± 0.3^b^	112 ± 1^b^	189 ± 1^b^	7.48 ± 0.4^a^	41.71 ± 1.0^a^	83.0 ± 2.5^b^	18.6 ± 1.5^b^	< 0.01	3:7 < 1:9, 2:8 < 1:9
Rice bran wax oleogel 1:9	2.58 ± 0.2^a^	1.3 ± 0.1^a^	4.2 ± 0.4^a^	113 ± 2^ab^	191 ± 2^a^	16.16 ± 0.8^a^	42.59 ± 1.2^a^	80.0 ± 3.0^b^	38.6 ± 2.0^a^	< 0.05	3:7 < 1:9, 2:8 < 1:9

Superscript letters denote significant differences among treatments according to ANOVA followed by Tukey’s HSD test (*p* < 0.05).

#### 3.1.2 Peroxide Value (PV)

Peroxide value (PV) is a major index of lipid oxidation, which measures the amount of hydroperoxides generated in the first stage of the oxidation rancidity process. The peroxide value of chickpea seed oil was 2.1 meq O_2_/kg and the peroxide values of the RBW‐based oleogels ranged from 1.8 (3:7), 3.5 (2:8) to 4.2 (1:9) depending on the mixture of wax. The results indicate unambiguously that higher rice bran wax (RBW) additions give more stable oxidative behavior whereas lower wax loadings lead to increased oxidation susceptibility. Among all the oleogels, the 3:7 oleogel, with the highest wax content, showed the lowest PV and indicates that the crystalline matrix, formed by RBW, efficiently entraps the oil and prevents the oxidation from the oxygen and from other pro‐oxidant factors. This is consistent with the results of Cho et al. (2023) that favored that biphasic edible gels with high content of RBW and soybean oil presented low peroxide values (< 3 meq/kg) and protected oxidative mechanisms due to the structured network during the storage conditions. On the other hand, the 1:9 blend presented a much higher PV value (4.2), borderline or above the restrictiveness of the quality control limits values allowed for edible oils (which are, in general, lower than 5 meq O_2_/kg). The lower wax concentration in this composition probably leads to a weaker gel structure with increased oil surface exposure and thus a faster oxidative process. Such a protective effect of the higher amounts of gelator content has already been reported by Ankaraligil and Aydeniz‐Guneser (2024), who demonstrated that increasing wax fraction in rice bran wax–canola oil oleogels resulted in a significant decrease in PV values during frying and storage. Moreover, Hwang et al. (2018) also reported that rice bran wax oleogels containing 3% rice bran wax had much slower oxidation rates than bulk oil, which supported the conclusions that oleogelation could be a strategy for improving oxidative stability in unsaturated oil systems.

#### 3.1.3 Iodine Value (IV)

Iodine value (IV) is a measure of the degree of unsaturation of fats and oils as determined by the amount of iodine that is absorbed by the double bonds of fatty acids. The IV of chickpea seed oil was 114 and the IVs of oleogels formulated using RBW were slightly lower: 110 (3:7), 112 (2:8), and 113 (1:9). These results show that the oleogelation process with rice bran wax slightly decreases the overall unsaturation of the oil matrix, especially for samples with more wax content. The slight decrease in IV of 3:7 and 2:8 formulations may be due to the presence of rice bran wax that consists predominantly of long‐chain saturated esters and alcohols and consequently, dilutes the total unsaturation in the gel. According to Hwang et al. (2025), rice bran wax oleogels prepared with different oils with decreasing level of unsaturation showed that IV was negatively correlated with the strength of the gels, substantiating that the introduction of wax decreases the degree of unsaturation and improves physical stability. Further, Choudhary et al. (2015) observed decreased IV of a mixture of rice bran oil with highly saturated fats (palm‐olein), in fresh as well as fried oils and proved the impact of saturated lipids in changing the IVs. Indeed, the 1:9 oleogel was more IV hanging to the pure chickpea oil (113 vs. 114) because of a quite low wax content. This indicates minimal effect on unsaturation when using less wax, contributing to a fingerprint closer to native oil.

#### 3.1.4 Saponification Value (SV)

Saponification value (SV) is the number of milligrams of KOH required for the saponification of 1 g of fat or oil. It also gives an idea about the average molecular weight of fatty acids; a higher value of SV indicates the short chain fatty acids, and vice versa. In the present study, the SV of chickpea seed oil was found as 190, and the RBW based oleogels had 188 (3:7), 189 (2:8) and 191 (1:9) for 3:7, 2:8 and 1:9 ratios, respectively. Corresponding to these results, marginal differences in the native oil compared to the oleogels can be observed, indicating that the process of oleogelation using rice bran wax does not necessarily change the distribution of the fatty acid chain length of the system. The slight variation in SV is caused by the ingredients of the wax. Rice bran wax is made up mainly of long‐chain saturated esters and alcohols, typical of the greatest decrease in SV with the addition of oil. That response was correspondingly found in a study by Xu et al. (2024), in which the saponification index was barely affected by the 1%–3% addition of rice bran and the oil on oleogel form and also increased the solid content, demonstrating the diluent effect of high molecular weight esters. Interestingly, the 1:9 oleogel presented the highest SV (191), only slightly higher than that of chickpea pure oil. This could be because of a higher content of oil than wax in the formula leading to more of average chain length reversion, the same as the original triglycerides. In the case of the 3:7 oleogel, indicating there are more long chain RBW esters decreasing the number of saponifiable functional groups per g of sample. These results are similar to those of the study by Limpimwong et al. (2017), who also reported limited changes in SV to RBW–rice bran oil oleogels stating that the functional gelation by waxes occurs without significant chemical transformation of the fatty acid profile.

#### 3.1.5 Determination of Fatty Acid Composition

**FIGURE 3:**
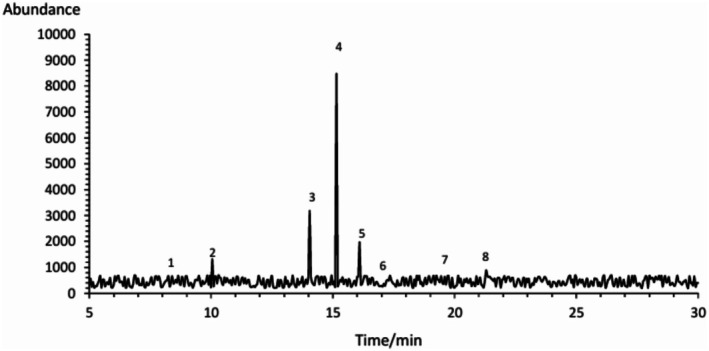
GC‐FID chromatogram of chickpea seed oil showing fatty acid methyl ester (FAME) peaks identified by retention time.

The gas chromatography analysis of chickpea ( *cicer arietinum* ) seed oil depicted the presence of fatty acid profile rich in unsaturated fatty acids, which is mainly represented by linoleic acid (C18:2, ω‐6) at 53% approximately, oleic acid (C18:1, ω‐9) around 20% and linolenic acid (C18:3, ω‐3) about 12%. In combination, these unsaturated account for greater than 85% of the total fatty acid content of chickpea oil, underscoring the potential for chickpea oil as a source of EFA. This formulation places the chickpea oil within the context of PUFA‐rich vegetable oils such as sunflower and safflower, which are well noted for their cardioprotective effects based on the levels of linoleic acid they contain. The predominance of the polyunsaturated over saturated fatty acids is consistent with present recommendations for reducing the risk of developing atherosclerosis by substituting unsaturated fat for saturated fat. The oleic acid content, a monounsaturated fatty acid with an already established reputation in providing oxidative stability and reduction on low‐density lipoproteins, is considered as a positive transportation medium not just as a source of macro and micronutrients but also of shelf‐life. In addition, it contains a fair amount of alpha‐linolenic acid (ALA) because a small concentration of ω‐3 is desirable for its anti‐inflammatory properties and fetus's neural, as well as cardiovascular health. Recent studies including that of Salaria et al. (2023), also rep‐portal ted similar FAs in chickpea varieties that were grown in Sudan, which testifies to having these similarities of nutritional nature even under different growing conditions. Curiously, long‐chain saturated fatty acids including behenic (C22:0), arachidic (C20:0), and lignoceric (C24:0) were also present at lower but apparent levels within this analysis. These long‐chain fatty acids, which do not increase cholesterol, are also known to influence the physical properties of lipid including crystallization and melting properties. The fact that either in low percentages they are still present indicates that they can be classified as “semi‐drying” oil, which is necessarily beneficial both for nutritional and for technofunctional exploitation like structuring into oleogels. The saponfiable fraction, which is rich in these fatty acids, predominantly in the form of triglycerides, hints the oleogelicity of the oil with solid fact rebulac, such as, rice bran wax (RBW). Its high unsaturation is advantageous to bioactivity and health functionality, while its saturated fraction is potentially useful in network formation and oxidative stability in gel systems (Dimakopoulou‐Papazoglou et al. 2023). Tusé et al. (2024) also confirmed the nutritive potential of such oils in vegetable‐based meal plans, and stated that the rich unsaturated profile of chickpea oil has a significant impact on lowering of LDL cholesterol and amelioration of metabolic parameters, emphasizing its role as a functional food component. To conclude, GC‐FID analysis of chickpea seed oil suggests its identity as a nutritionally rich lipid source with high PUFA (supplied by Linoleic and Oleic acids), moderate omega‐3 and trace long chain saturates and therefore it could serve as a functional food source as well as a nutrient supplement. These properties not only enhance its health‐beneficial effects, but also give it the structural fit required for incorporation into wax‐was oleogels targeted for fat reduction and shelf‐stable applications. The chromatographic profile illustrating these FAME peaks and their retention times is presented in Figure 3.

### 3.2 Functional Property Analysis of Oleogels

#### 3.2.1 Binding Capacity and Migration Ability

**FIGURE 4:**
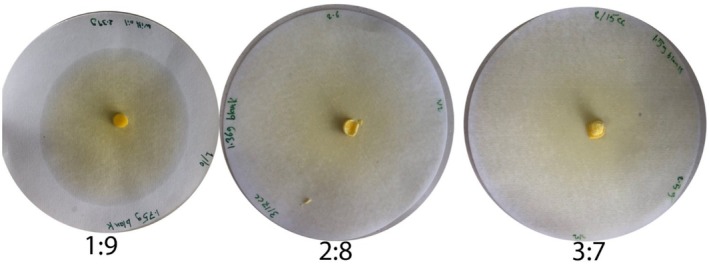
Oil migration assessment of chickpea oil‐based oleogels prepared with rice bran wax (RBW) at different ratios: 1:9, 2:8, and 3:7 (RBW: Oil).

Oil binding capacity (OBC) is an important functional characteristic of oleogels and refers to the holding power of the oleogel on the oil within the gel network, whereas oil migration describes the release of oil from the gel during storage. The visual differences in migration halos among the three oleogel formulations are shown in Figure 4. Pure liquid oil The OBC was not determined in the chickpea seed oil because this oil is free flowing. Among the oleogels, the OBC value was highest in the 3:7 formulation (96.0%) followed by 2:8 (83.0%) and 1:9 (80.0%). On the contrary, the higher with the lower wax content 16.4% (3:7), 18.6% (2:8) and 38.6% (1:9). These findings reveal that the concentration of rice bran wax (RBW) is essential for the construction of a close cross‐crystal network to immobilize oil. The higher performance of the 3:7 formulation is in agreement with the observations of Jones et al. (2022) that at 4% in corn oil, RBW‐structured oleogels contained > 99% of added oil, which reflected the presence of strong spherulitic and platelet like crystal structures. In addition, Blake (2015) explained that more compact networks with lower pore fractions are linked with higher oil retention and lower migration. Her research revealed that by following the principles of shear‐induced crystallization and slow cooling, the structural homogeneity of RBW oleogels is enhanced followed by the successful adhesion and entrapment of oil into the micro pores. The rapid increase in oil migration in 1:9 sample is indicative of disruption of the crystalline matrix as a result of lower wax concentration bringing about bigger voids and inadequate gel strength. this was also noted by Ramadhan et al. (2024) the oil binding capacity differed greatly within the marine oleogel systems when wax content was lower to 5%, and showed poor structure and high oil expression.

#### 3.2.2 Texture Analysis

**FIGURE 5:**
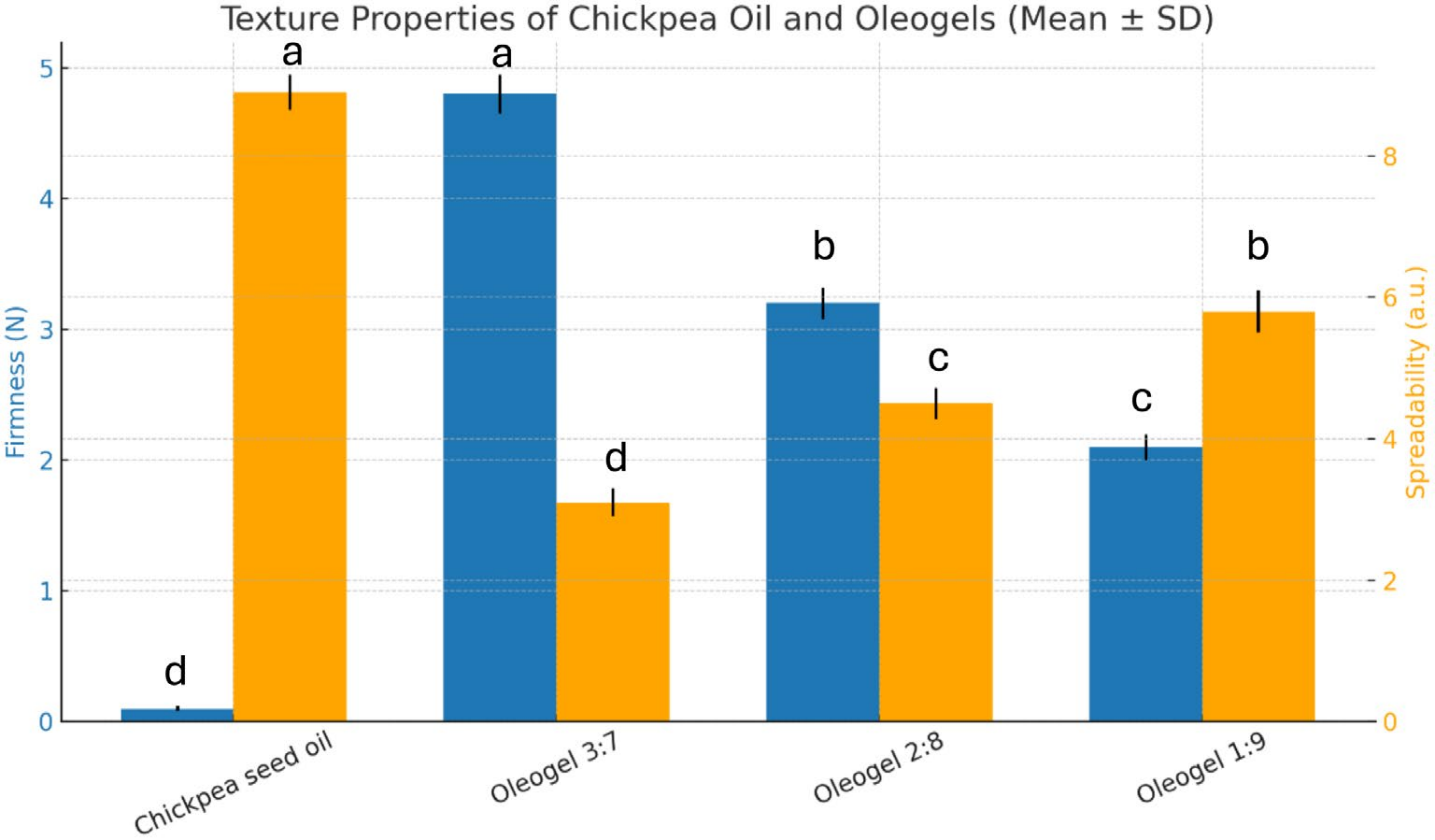
Texture properties of chickpea seed oil and oleogels with different oil‐to‐structurant ratios, expressed as firmness (N) and spreadability (a.u.) (mean ± SD, *n* = 3). Different superscript letters above bars indicate statistically significant differences among treatments within the same parameter (one‐way ANOVA followed by Tukey's HSD test, *p* < 0.05).

Texture is a critical parameter for evaluating the structural functionality of oleogels, especially when used as fat replacers in food products. In this study, two primary textural attributes were measured: firmness and spreadability. The comparative textural behavior of chickpea oil and its oleogel formulations is illustrated in Figure 5. Firmness represents the resistance of the oleogel to deformation under pressure (in Newtons), while spreadability reflects the ease with which the gel can be applied or manipulated, where lower values indicate better spreadability. The texture analysis revealed that the firmness of the oleogels increased with higher wax concentration, ranging from 2.1 N in the 1:9 formulation to 4.8 N in the 3:7 formulation. In contrast, the control chickpea seed oil had negligible firmness (0.1 N), confirming its liquid state. Spreadability values followed the opposite trend: the 3:7 formulation had the lowest spreadability (3.1 a.u.), while 1:9 and 2:8 had progressively higher values (5.8 and 4.5 a.u., respectively). Chickpea oil, being a free‐flowing liquid, showed the highest spreadability (8.9 a.u.). These findings are also well‐supported by previous works carried out on wax‐based oleogels. For example, Jones et al. (2022) reported that rice bran wax (RBW) could produce stable and hard (oleo)gels even at low concentrations, and have a much better strength and oil binding capacity than carnauba wax. Firmness values as high as 66.9 g (0.66 N) for only 2% RBW in corn oil oleogels were presented in the study, underlining the excellent structuring potential for this type of oleogel. Similarly, Ferdaus et al. (2022) found that the firmness and spreadability of peanut butter samples structured with rice bran wax oleogels were significantly higher compared to those using hydrogenated oils. Their report also showed that RBW‐based oleogels exhibited texture properties superior to similar commercial products and could be a potential ingredient in food formulations with a semisolid texture need (Ferdaus et al. 2022). Moreover, Pang et al. (2022) observed a more compact crystal network and enhanced hardness in cookie shortening replacers when RBW content was increased. The best‐designed composition of theirs (6 wt. %) formed a very strong gelled structure with 15.75 N of hardness indicating a significant relationship at wax concentration levels and gel strength in different food systems (Pang et al. 2022). Collectively, textural results in this study supported that the increment of rice bran wax resulted in increased gel strength as well as decreased spreadability of the jelly product, making the jelly a firmer and more stable product. These properties are especially useful for replacing saturated fats in spreads, bakery products and meat analogues, which require both a structure and functionality.

#### 3.2.3 Differential Scanning Calorimetry (DSC)

**FIGURE 6:**
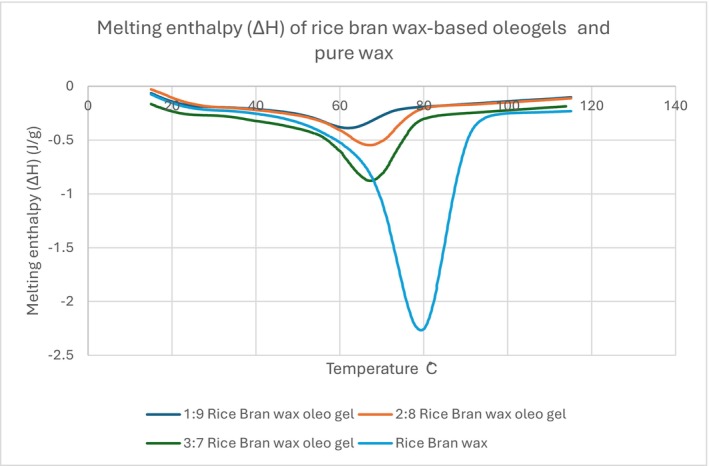
Melting enthalpy (Δ*H*) of rice bran wax oleogels and the pure wax as determined by DSC.

The melting profile of rice bran wax (RBW) oleogel was studied through Differential Scanning Calorimetry (DSC), with thermograms revealing the presence of well‐defined endothermic transitions for each formulation. The variations in melting enthalpy (Δ*H*) among the pure wax and oleogel samples are illustrated in Figure 6. Pure rice bran wax had the most intense and sharp melting peak at approximately 77°C, with a melting enthalpy of −2.5 J/g, indicating its higher crystallinity and thermal stability. This is in agreement with those of Wijarnprecha et al. (2018), who also described that the presence of a higher focus of RBW led to needle‐like wax crystals and orthorhombic subcell packing structures, thereby increasing the thermal resistance and the network integrity. When the wax level decreased in the oleogel samples (from 3:7 to 1:9), the melting peaks became wider, less defined, and at lower temperatures. The 3:7 sample still had a sharp melting peak at ~73°C, with a relatively large enthalpy, which means good structural integrity, although their peak temperatures and enthalpies were decreasing with the 2:8 and 1:9 treatments, since the chickpea oil has diluted the crystalline network. This decrease is consistent with Jones et al. (2022), who showed that even at low concentrations, RBW offers greater firmness and enthalpy compared to other waxes, due to its efficient crystal packing and strong network formation. In addition, Tavernier et al. (2017) have emphasized the role of the wax crystallization kinetics and confirmed that a high‐melting wax (such as RBW) will form more cohesive and thermally stable structures than a low‐melting one. Their DSC and XRD results also confirmed that both stepwise crystallization and homogeneity of wax networks were closely related to melting behavior and gel strength (Tavernier et al. 2017).

#### 3.2.4 X‐Ray Diffraction (XRD) Analysis

**FIGURE 7:**
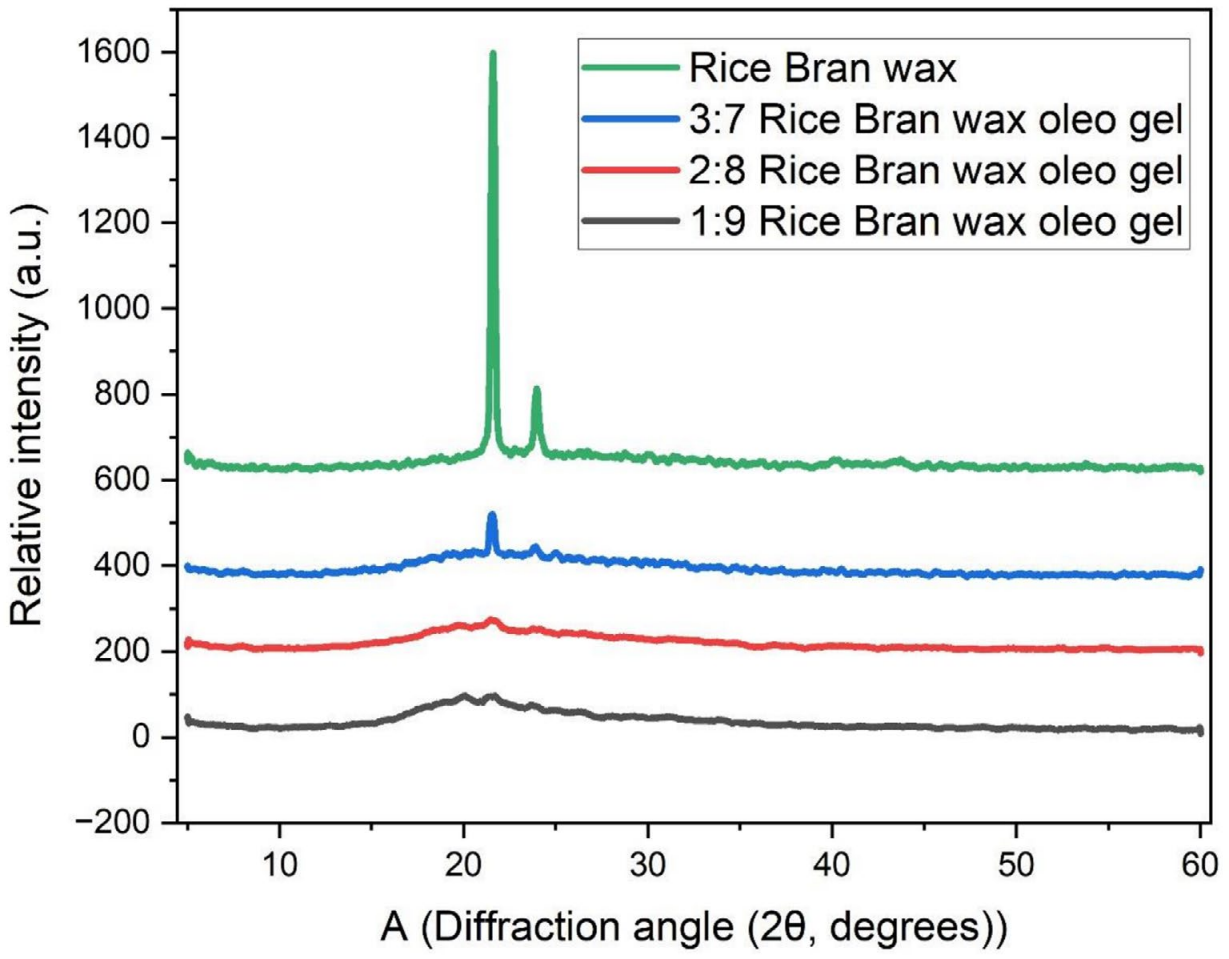
X‐ray diffraction (XRD) patterns of raw rice bran wax and chickpea oil (containing 3:7, 2:8, and 1:9 wax and oil ratios) based oleogel formulations.

The X‐ray diffraction (XRD) diffraction mutually patterns for neat rice bran wax and its corresponding oleogel systems (3:7, 2:8 and 1:9) show the influence of the wax amount into gel characteristics of the systems. The XRD profiles of all samples are shown in Figure 7. Characteristics peaks of rice bran wax are observed in the diffractograms around 2θ = 20°–23°, being those peaks representative of long chain crystalline materials. These peaks are attributed to an orthorhombic subcell packing, which has been reported as a normal structure in fully crystallized natural waxes (Wijarnprecha et al. 2018). Of the four samples, pure RBW showed the most dominant and well‐defined peaks, suggesting a well‐ordered and crystalline phase. With the decrease of wax concentration in the oleogel samples, the imitation of the peak intensity and sharpness decreased constantly, too. The 3:7 oleogel exhibited intermediate crystallinity indicating a well‐defined crystal network able to support gel stability. On the other hand, the 2:8 and 1:9 samples were characterized by much weaker and broader peaks, indicating the lower crystallinity and the higher degree of amorphousness. This is due to the wax dilution by chickpea oil, which disrupts crystal packing and results in a decrease of long‐range molecular order. These results are consistent with research like Tavernier et al. (2017) who reported that decreased wax content led to lower crystalline space packing order because of lack of wax‐wax interactions in the liquid oil. Furthermore, Jones et al. (2022) showed that RBW is superior to other waxes in preserving the crystalline structure in oleogels, especially at higher concentrations.

#### 3.2.5 Elemental Analysis Using CHN Analyzer

**FIGURE 8:**
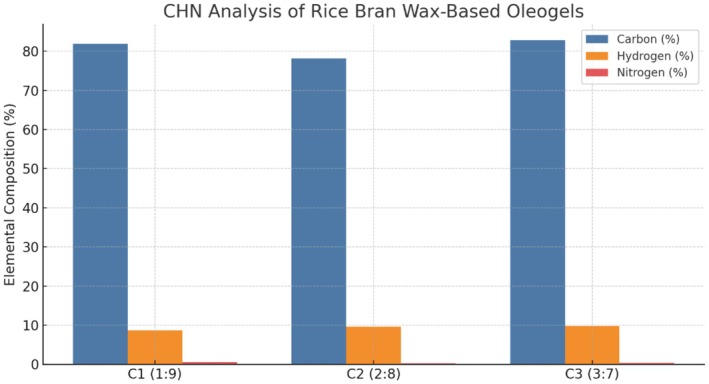
Elemental composition (Carbon, Hydrogen, Nitrogen) of rice bran wax‐based oleogels with varying wax‐to‐oil ratios (C1: 1:9, C2: 2:8, C3: 3:7).

CHN elemental analysis of the rice bran wax–based oleogels demonstrated significant differences in carbon, hydrogen, and nitrogen concentration associated with the increasing of wax‐to‐oil ratio. The comparative elemental composition of the three formulations is shown in Figure 8. It can be observed that all three oleogels displayed high carbon composition (78.19%–82.81%), presumably attributed to the mostly organic and hydro‐carbonic‐based chickpea oil and rice bran wax. The C3 (3:7) formulation showed the highest carbon content, consistent with higher wax content of this formulation, because RWB consists mainly of LCFA alcohols and wax esters with high carbon content. The hydrogen contents were close together for all formulations (between 8.69% and 9.79%), and similar to before showed a slight rise for the C3 oleogel and hence higher wax content. These values are also in the typical range of lipids and demonstrate the oleogels are an oleogel rich in hydrocarbons. All samples contained very limited amounts of nitrogen (0.27%–0.51%), indicating that protein contamination or remaining seed biomass was minimal and the oil had been effectively extracted and purified prior to gelation. The total elemental composition totaled 88.10%–92.95% of sample weight indicating purity and reproducibility of the formulations. These findings are in agreement with those published by Zhu et al. (2023) were analyzed, oleogels forms by natural waxes and oils and similar high content in carbon and hydrogen and absence of nitrogen was found, pointing to the clean, non‐proteinaceous lipidic nature. Moreover, Winuprasith and Suphantharika (2015) reported that nitrogen in oleogels systems is usually lower than 1% (unless, as mentioned before, proteins or amino acids are intentionally added) confirming the good and non protein nature of your oleogels.

#### 3.2.6 Fourier Transform Infrared Spectroscopy (FTIR)

**FIGURE 9:**
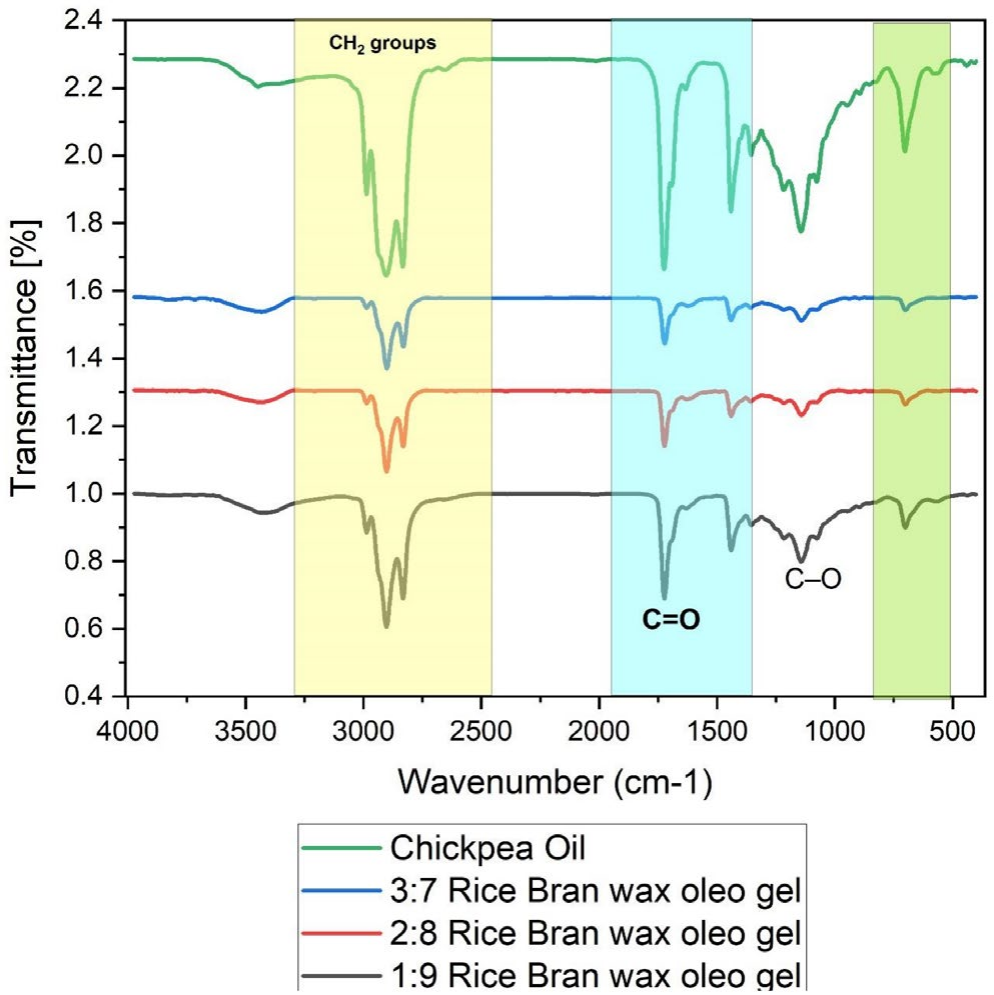
FTIR spectra of chickpea oil and rice bran wax‐based oleogels (ratios 3:7, 2:8, and 1:9).

The chemical structure and functional group interaction of chickpea oil and RBW‐based olegel formulations at (3:7, 2:8 and 1:9) wax to oil ratio were investigated using FTIR spectroscopy. The FTIR spectra of all samples are shown in Figure 9. Transmittance spectra in the 4000–500 cm^−1^ region show characteristic peaks for fatty acid chains, ester linkages and hydrocarbons, and spectral similarities and differences give an indication of the interaction at the molecular level during gelation. All the samples (including the native chickpea oil) showed intense absorption bands at 2920 and 2850 cm^−1^ corresponding to the asymmetric and symmetric stretching of the CH_2_ groups—typical of long hydrocarbon chains which are present in both the triglycerides and wax esters. These two peaks were consistent for all oleogel samples, revealing that oleogelation did not induce any chemical change to the hydrocarbon core structure. One high intensity peak at around 1740 cm^−1^ may be assigned to the C=O stretching vibration of the ester groups, representing the existence of triglycerides and wax esters in both the oil and wax components. The oleogel samples also exhibited a small decrease in the peak intensity observed at 1740 and 1460 cm^−1^, which are indicative of slight physical interactions due to hydrogen bonding or van der Waals resulting in gel network structure. This is in corroboration with the findings of Dhal et al. (2023), the FTIR spectra of soy wax and rice bran oil‐based oleogels are similar to their raw materials, indicating that gelation was mainly controlled through non‐covalent physical interactions, instead of chemical modifications. Crucially, the lack of new peaks or significant changes in the spectra indicates that no new covalent bonds were formed during oleogel development. This was also confirmed by Saffold and Acevedo (2021) who observed that the FTIR spectrum of oleogel loaded bigels displayed the existing peaks of both phases without the appearance of new peaks, suggesting the preservation of native functional groups upon oleogelation. Additionally, the oleogels exhibited new bands in the range of 1100–1000 cm^−1^, attributed to the C–O stretching of ester and alcohol and such bands were more pronounced in the higher wax oleogel formulations. This holds the suggestion that higher enzymes which resulted in higher wax might provide a more ordered network of the esterified structures, an example by facilitating higher mechanical properties with the same chemical structure.

#### 3.2.7 Antioxidant Activity (DPPH and ABTS)

**FIGURE 10:**
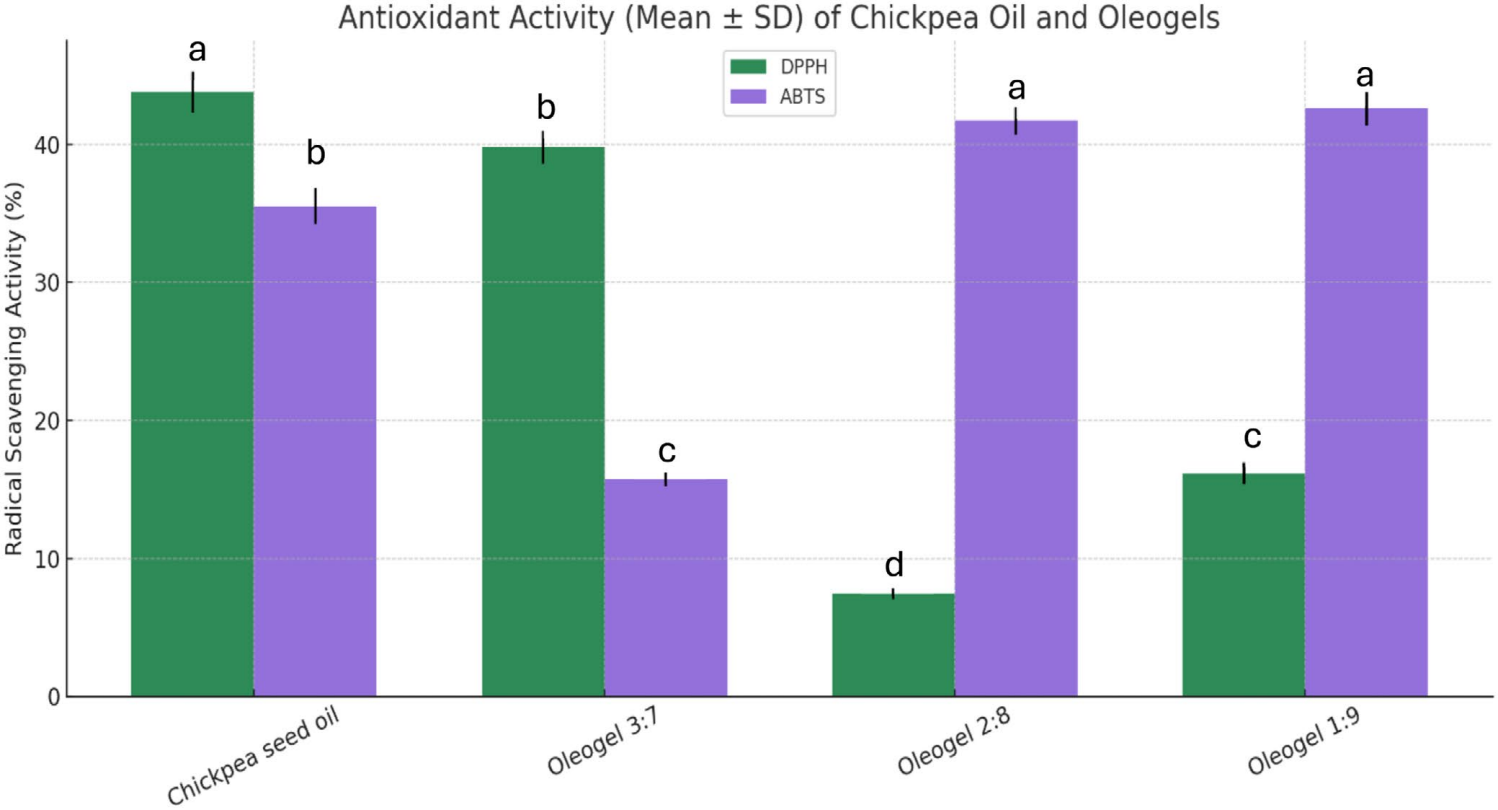
Antioxidant activity of chickpea seed oil and oleogels determined by DPPH and ABTS radical scavenging assays (mean ± SD, *n* = 3). Different superscript letters above bars indicate statistically significant differences among treatments within the same assay (one‐way ANOVA followed by Tukey's HSD post hoc test, *p* < 0.05).

Antioxidant activity was evaluated using two radical scavenging assays: DPPH (2,2‐diphenyl‐1‐picrylhydrazyl) and ABTS (2,2′‐azino‐bis(3‐ethylbenzothiazoline‐6‐sulfonic acid)). These assays reflect the capacity of the samples to neutralize free radicals, a crucial property for oxidative stability in lipid‐based systems. A comparison of the DPPH and ABTS activities of chickpea oil and the oleogel formulations is shown in Figure 10. Chickpea seed oil exhibited a DPPH scavenging activity of 43.79% and ABTS activity of 7.48%, indicating inherent antioxidant potential due to its phenolic and unsaturated fatty acid content. Among the oleogels, the 3:7 formulation demonstrated the highest ABTS activity (41.71%), followed by 1:9 (42.59%), while 2:8 showed moderate levels (16.16%). Among oleogels, the activity of 2:8 was the highest (39.82%) as DPPH activity followed by 3:7 (35.53%) then 1:9 (15.75%). The difference in antioxidant response may be associated with the structural interaction of RBW–chickpea oil. The 3:7 and 2:8 formulations have higher RBW concentrations, which are filled with unsaponifiable matter—such as γ‐oryzanol, tocopherols, phytosterols—recognized by their antioxidant activity. Sahu et al. (2020) found that oleogels fortified with unsaponifiables from rice bran oil distillate had higher antioxidant capacity (DPPH and ABTS) than control oils. Moreover, the research of Xiao et al. (2020) reported strong DPPH and ABTS scavenging activities from rice bran protein hydrolysates, especially those containing large amounts of glutamic acid and hydrophobic amino acid, suggesting the potential of rice bran as a natural source of multifunctional antioxidant. Higher ABTS activity in 1:9, compared to lower DPPH value, may be due to hydrophilic vs. lipophilic radical scavenging properties influenced by oil–wax ratio. These results are consistent with the report of Ham et al. (2016) who found that unsaponifiable matter, obtained by saponification from rice bran, exhibited better antioxidant activities, particularly in ABTS assays, owing to the rich contents of bioactive lipid‐soluble substances.

### 3.3 Antimicrobial Activity

**FIGURE 11:**
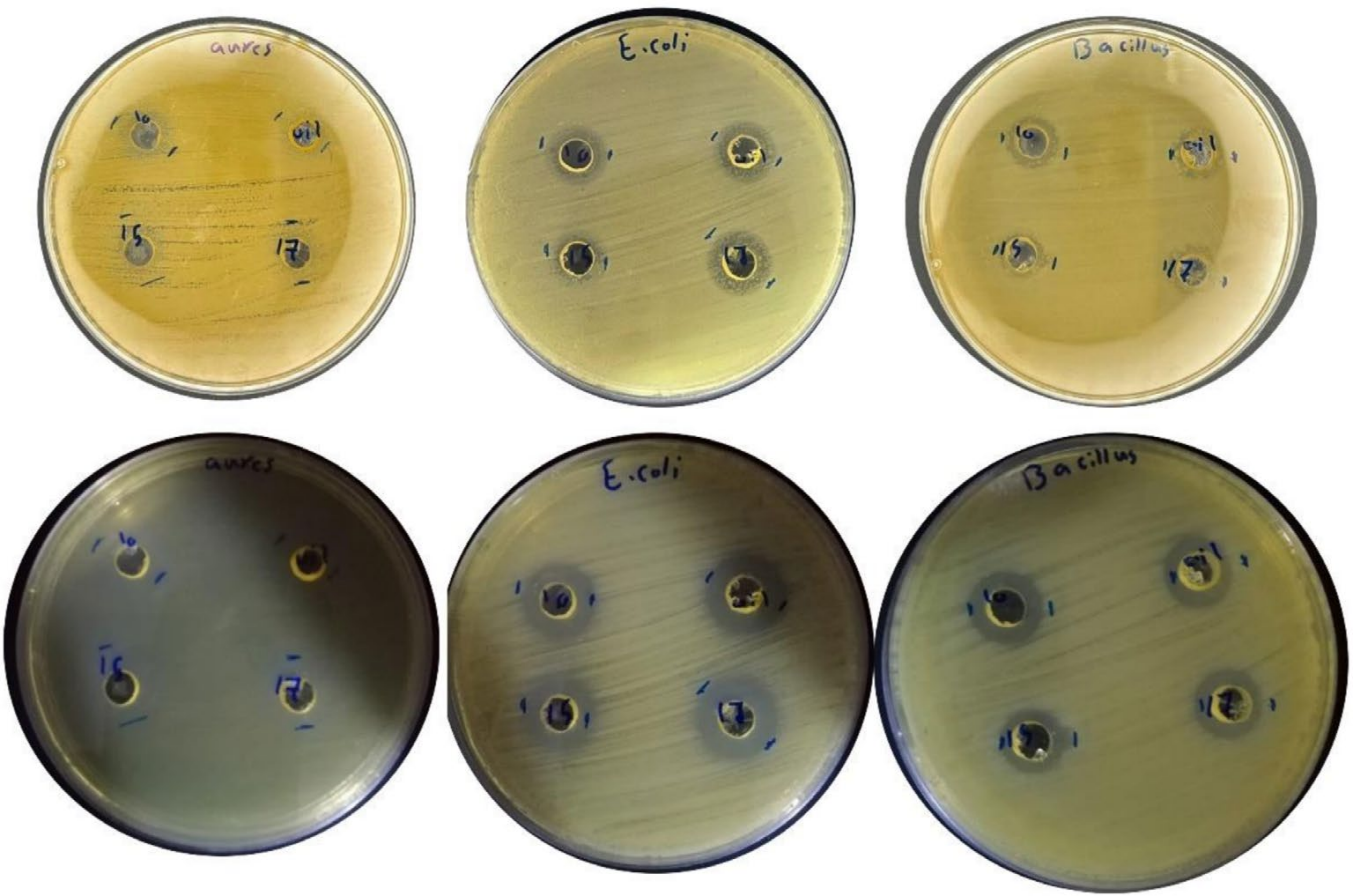
Agar well diffusion assay showing zones of inhibition for chickpea oil and rice bran wax‐based oleogels (1:9, 2:8, 3:7) against three bacterial strains: *Staphylococcus aureus* (left), *Escherichia coli* (middle), and *Bacillus subtilis* (right).

The antimicrobial evaluation of chickpea oil and rice bran wax (RBW) oleogels was conducted against three representative foodborne and clinical pathogens: *Bacillus subtilis, Escherichia coli*, and *Staphylococcus aureus* . The inhibition zones observed for chickpea oil and the three oleogel formulations against the tested bacteria are shown in Figure 11, while the corresponding quantitative data and statistical comparisons are summarized in Table 3. The results showed that pure chickpea oil consistently demonstrated the highest average inhibition zones against all tested microorganisms: 14.47 mm (Bacillus), 15.62 mm ( *E. coli* ), and 13.11 mm ( *S. aureus* ). This highlights the inherent antimicrobial properties of chickpea oil, possibly due to its rich content of unsaturated fatty acids and minor bioactive components such as polyphenols and tocopherols. Among the oleogels, a clear wax concentration‐dependent effect was observed. The 1:9 RBW oleogel formulation showed inhibition values very close to the oil alone, especially against *E. coli* (15.20 mm) and *S. aureus* (13.08 mm), suggesting that low wax concentrations maintain good diffusion of active compounds. However, increasing the wax content progressively reduced inhibition zones, as seen in 3:7 RBW oleogel where values dropped to 12 mm (Bacillus), 11.3 mm ( *E. coli* ), and 10.9 mm ( *S. aureus* ). These findings are supported by Kabploy et al. (2022), who found that both crude extract and oil from rice bran exhibited significant antibacterial activity against *Bacillus cereus* , *S. aureus* , and *E. coli* , with inhibition zones and MICs suggesting strong bioactivity at low concentrations. They attributed this activity to phytosterols, tocopherols, and γ‐oryzanol present in rice bran oil and wax, which are known for disrupting bacterial membranes and oxidative metabolism. Notably, the oleogel structure plays a critical role in modulating antimicrobial effectiveness. As shown in studies by Wijarnprecha et al. (2018) and Yao et al. (2021), higher concentrations of RBW lead to denser crystal networks, which while improving textural stability, may restrict the release or diffusion of antimicrobial constituents. This explains the inverse relationship observed between wax content and inhibition zone size.

**TABLE 3:** Antimicrobial activity data for chickpea oil and RBW‐based oleogels.

Microorganism	Oil (mean ± SD)	Oleogel 1:9 (mean ± SD)	Oleogel 2:8 (mean ± SD)	Oleogel 3:7 (mean ± SD)	*p* (ANOVA)	Post hoc test (Tukey's HSD)
*Bacillus subtilis*	14.47 ± 0.3^a^	13.99 ± 0.2^a^	13.10 ± 0.4^b^	12.00 ± 0.5^c^	< 0.05	3:7 < 1:9, 3:7 < 2:8
*Escherichia coli*	15.62 ± 0.2^a^	15.20 ± 0.3^a^	12.80 ± 0.4^b^	11.30 ± 0.3^c^	< 0.01	3:7 < 1:9, 3:7 < 2:8
*Staphylococcus aureus*	13.11 ± 0.3^a^	13.08 ± 0.2^a^	12.50 ± 0.3^b^	10.90 ± 0.3^c^	< 0.05	3:7 < 1:9, 3:7 < 2:8

*Note:* Zone of inhibition (mm) of different treatments against tested microorganisms. Values represent mean SD.

Superscript letters denote significant differences among treatments according to ANOVA followed by Tukey’s HSD test (*p* < 0.05).

## 4 Conclusion

This study successfully demonstrated the potential of chickpea ( *Cicer arietinum* L.) seed oil as a base for functional oleogels structured with rice bran wax (RBW). The extracted oil exhibited a favorable fatty acid profile, dominated by polyunsaturated fats—particularly linoleic acid (52.99%) and oleic acid (19.92%)—and showed good oxidative properties (acid value: 2.7, peroxide value: 2.1 meq O_2_/kg, iodine value: 114). Oleogels were prepared at RBW‐to‐oil ratios of 3:7, 2:8, and 1:9, and their functional performance was evaluated through a series of physicochemical, antioxidant, and antimicrobial analyses. The 3:7 formulation outperformed others in terms of oil binding capacity (96.0%), firmness (4.8 N), and oxidative stability (acid value: 1.1, PV: 1.8 meq O_2_/kg). Differential Scanning Calorimetry (DSC), X‐ray Diffraction (XRD), and Fourier Transform Infrared Spectroscopy (FTIR) confirmed that RBW formed a strong crystalline network through physical interactions, without chemical modifications. Antioxidant testing showed that the 2:8 formulation had the highest ABTS inhibition (41.71%), while 3:7 performed best in DPPH inhibition (39.82%). Furthermore, all formulations showed antimicrobial activity, with chickpea oil and 1:9 oleogels showing larger inhibition zones against *E. coli* and *Staphylococcus aureus* . Overall, the study confirmed that chickpea seed oil–RBW oleogels are a promising plant‐based alternative to conventional solid fats, offering structural stability, oxidative resistance, and bioactivity for use in functional foods.

## Author Contributions

**Farhang Hameed Awlqadr:** methodology (equal), project administration (equal), resources (equal), supervision (equal), writing – original draft (equal), writing – review and editing (equal).Othman Abdulrahman Mohammed: methodology (equal), resources (equal). **Syamand Ahmed Qadir:** methodology (equal), project administration (equal), resources (equal), supervision (equal), validation (equal), writing – original draft (equal), writing – review and editing (equal). **Ako Mahmood Qadir:** conceptualization (equal), data curation (equal), investigation (equal), methodology (equal), resources (equal), visualization (equal). **Aryan Mahmood Faraj:** conceptualization (equal), data curation (equal), formal analysis (equal), methodology (equal), validation (equal). **Khalied Arab:** writing‐review and editing (equal).

## Data Availability

The data supporting the findings of this study are available from the corresponding author upon reasonable request. All raw and processed data generated or analyzed during the current study, including physicochemical analyses, chromatograms, and spectral datasets, are stored at Halabja Technical College, Sulaimani Polytechnic University, and can be made available upon request.
